# Influencing factors and pathways of benefit finding in young and middle-aged patients with first-episode acute myocardial infarction and their spouses: a path analysis

**DOI:** 10.3389/fpsyg.2026.1852033

**Published:** 2026-07-01

**Authors:** Ru Wang, Fu Zhang, Hui You, Xiaoyan Li, Wentong Hui, Lidong Ma

**Affiliations:** Department of Cardiology, General Hospital of Ningxia Medical University, Yinchuan, Ningxia Hui Autonomous Region, China

**Keywords:** actor-partner interdependence model, benefit finding, first-episode AMI patients, resilience, spouses, young and middle-aged

## Abstract

**Background:**

Young and middle-aged patients with first-episode acute myocardial infarction (AMI) and their spousal face significant psychological challenges, yet existing research often overlooks their dyadic interdependence in the process of benefit finding.

**Aim:**

To investigate the levels of benefit finding in young and middle-aged patients with first-episode acute myocardial infarction and their spouses, to identify the influencing factors, and to analyze the pathways among these factors.

**Methods:**

A cross-sectional study was conducted among 212 dyads of young and middle-aged patients with first-episode acute myocardial infarction and their spouses. Data were collected using a set of validated scales to measure benefit finding, psychological resilience, coping styles, and family function. Data analyses encompassed descriptive statistics, Pearson correlation, multiple linear regression, and path analysis via the Actor-Partner Interdependence Model (APIM) to examine the actor and partner effects within the dyads.

**Results:**

Benefit finding scores were (64.77 ± 13.05) for patients and (65.65 ± 12.87) for spouses. Multiple regression revealed that patients’ benefit finding was significantly associated with their own education, comorbidities, number of stents, resilience, positive coping, and their spouse’s resilience. For spouses, their benefit finding was associated with their own education, age, resilience, family function, as well as the patient’s age and comorbidities (all *p* < 0.05). The Actor-Partner Interdependence Model (APIM) further showed significant actor effects: both patients’ and spouses’ resilience and positive coping positively predicted their own benefit finding. Moreover, spouse’s family function exhibited a significant actor effect on their own benefit finding. Regarding partner effects, both patients’ and spouses’ resilience positively predicted each other’s benefit finding.

**Conclusion:**

Benefit finding in both patients and spouses is moderate-low and influenced by multiple individual and dyadic factors. Interventions should target resilience, coping strategies, and family function from a dyadic perspective to enhance benefit finding.

## 1 Introduction

Acute myocardial infarction (AMI), a severe manifestation of coronary heart disease (CHD), is associated with high mortality and disability rates (Ibanez et al., 2018). China currently has approximately 11.39 million CHD patients, with young and middle-aged individuals accounting for 5–30% of AMI cases (Tang et al., 2023; Wang et al., 2023). Percutaneous coronary intervention (PCI) is a primary method for treating CHD and achieving coronary revascularization.

As the core workforce and primary financial providers for their families, young and middle-aged AMI patients face significant lifestyle disruptions following their illness, including long-term medication use and work interruptions or leave. These changes impose substantial economic and psychological burdens on their families (Zhang Q. et al., 2024). Spousal, who typically bear the primary caregiving responsibilities, play a crucial role in patients’ disease management, treatment decisions, self-care practices, and daily living activities (McHorney et al., 2021). The illness not only causes physical and psychological distress for patients but also negatively impacts spouses’ mental health, leading to increased depression, heightened caregiving burden and stress, and reduced quality of life (Cai et al., 2020).

With the emergence of positive psychology, researchers have observed that individuals can derive meaning and positive experiences from adverse life events such as illness or trauma, a phenomenon termed “benefit finding” (Brand et al., 2016). Studies indicate that benefit finding can effectively alleviate psychological distress in both patients and spouses, promote proactive social support seeking, enhance psychological resilience, and reduce psychological suffering and caregiving burden (Jin et al., 2021; Sun et al., 2022; Wei et al., 2019). Furthermore, benefit finding can be strengthened through adaptive illness coping strategies and positive family relationships.

AMI patients and their spouses form a closely interdependent dyad, where changes in cognition, behavior, or emotion in one partner can readily influence the other (Lee et al., 2018). It is therefore plausible that the psychological resilience, illness coping strategies, and perceived family support of both patients and their spouses may influence their own and their partner’s experience of benefit finding. However, existing research primarily focuses on the patient perspective, often overlooking this inherent dyadic interdependence.

The Actor-Partner Interdependence Model (APIM) accounts for the interdependence within linked dyadic data by simultaneously analyzing actor effects (where an individual’s outcome is influenced by their own predictor variables) and partner effects (where an individual’s outcome is influenced by their partner’s predictor variables) (Uchmanowicz et al., 2022). This study employs the APIM framework to investigate the current status and influencing factors of benefit finding among young and middle-aged patients experiencing their first AMI and their spouses. Using a dyadic perspective, we will establish a structural equation model to elucidate the relationships and pathways among these influencing factors, with the findings aiming to inform the development of targeted interventions.

## 2 Methods

### 2.1 Participants

A convenience sample of patients who were admitted with acute myocardial infarction and underwent PCI between October 2024 and March 2025 was recruited from the cardiology departments of tertiary hospitals in Yinchuan. Of 278 eligible dyads, all 278 were invited to participate; 236 agreed (85.0% response rate), and 24 were excluded due to incomplete questionnaires, resulting in a final sample of 212 dyads with complete data (no missing data). Questionnaires were completed during hospitalization, typically 3–7 days after PCI.

### 2.2 Inclusion and exclusion criteria for the patients

Inclusion criteria: ① meeting the diagnostic criteria for acute myocardial infarction according to the 2025 ACC/AHA/ACEP/NAEMSP/SCAI Guideline for the Management of Patients With Acute Coronary Syndromes (Rao et al., 2025); ② Underwent coronary angiography and subsequent coronary stent implantation; ③ Aged 18–59 years (based on WHO age criteria); ④ Married, with their spouse serving as the primary caregiver; ⑤ Adequate communication and reading comprehension abilities; ⑥ Provided informed consent and agreed to participate in follow-up; ⑦ Complete clinical data available.

Exclusion criteria: ① Presence of unstable, life-threatening complications (e.g., severe organic heart disease, renal failure, malignancy); ② Severe cognitive impairment or psychiatric disorders; ③ Concurrent participation in other clinical intervention studies.

### 2.3 Inclusion and exclusion criteria for the spouse

Inclusion Criteria: ① Aware of the patient’s diagnosis; ② Normal comprehension and communication abilities.

Exclusion Criteria: ① Presence of unstable, life-threatening complications (e.g., severe organic heart disease, renal failure, malignancy); ② Severe cognitive impairment or psychiatric disorders; ③ Concurrent participation in other clinical intervention studies.

### 2.4 Sample size

The required sample size was determined based on the Actor-Partner Interdependence Model (APIM) specifications outlined by Kenny et al. (2006). With a desired statistical power of 0.80, a two-tailed significance level of 0.05, and an assumed actor-partner and residual correlation of 0.26, a minimum of 111 dyads is required to detect meaningful actor and partner effects. In the present study, a total of 212 patient-spouse dyads were recruited. Exceeding the minimum requirement and ensuring adequate power for the planned path analyses.

### 2.5 Measures

A general information questionnaire was developed by the researchers based on a literature review. It comprises two sections:

The patient questionnaire captured demographic and clinical information, including sex, residence, employment status, education level, medical payment method, monthly household income, number of children, number of comorbid chronic conditions, number of hospitalizations per year, number of stents implanted, and infarct location.

The spouse questionnaire captured demographic information, including sex, age, employment status, education level, and number of comorbid chronic conditions.

#### 2.5.1 Benefit finding scale (BFS)

Originally developed by Antoni et al. (2001) to assess benefit finding in cancer patients, the scale was cross-culturally adapted into Chinese by Liu et al. (2014). The scale comprises 22 items across six dimensions: Acceptance, Family Relationships, Worldview, Personal Growth, Social Relationships, and Health Behaviors. Items are rated on a 5-point Likert scale from 1 (“Not at all”) to 5 (“Very much”), with total scores ranging from 22 to 110. Higher scores indicate higher levels of benefit finding. The original scale’s Cronbach’s *α* was 0.950. In this study, Cronbach’s *α* coefficients were 0.941 for patients and 0.944 for spouses.

#### 2.5.2 Connor-Davidson resilience scale (CD-RISC)

Developed by Connor and Davidson (2003) and cross-culturally adapted by Wu et al. (2017), this scale measures an individual’s ability to cope with stressors. It contains 25 items across three dimensions: Optimism, Strength, and Tenacity. Items are rated on a 5-point Likert scale from 0 (“Never”) to 4 (“Always”), with total scores ranging from 0 to 100. Higher scores indicate greater resilience. The original scale’s Cronbach’s *α* was 0.910. In this study, Cronbach’s *α* coefficients were 0.949 for patients and 0.946 for spouses.

#### 2.5.3 Simplified coping style questionnaire (SCSQ)

Developed by Chinese scholar Xie (1998), this questionnaire assesses coping styles. It consists of 20 items forming two dimensions: Positive Coping and Negative Coping. Items are rated on a 4-point Likert scale from 0 (“Never adopt”) to 3 (“Often adopt”). Higher scores on the Positive Coping dimension indicate a greater tendency to use positive coping strategies. The scale’s Cronbach’s *α* coefficient was 0.900. In this study, Cronbach’s α coefficients for the positive and negative coping subscales were 0.898 and 0.905, respectively.

#### 2.5.4 Family APGAR index (FAI)

The scale was developed by Smilkstein et al. (1982). It includes 5 items representing five dimensions: Adaptability, Partnership, Growth, Affection, and Resolve. Items are rated on a 3-point Likert scale: “Hardly ever” (0 points), “Some of the time” (1 point), and “Almost always” (2 points). Total scores range from 0 to 10, interpreted as severe dysfunction (0–3), moderate dysfunction (4–6), or good family function (7–10). In this study, Cronbach’s *α* coefficients were 0.889 for patients and 0.890 for spouses.

### 2.6 Procedures

Prior to data collection, researchers received uniform training to standardize instructions and explanations and ensure familiarity with the survey instruments. During the formal survey phase, participants (patients and their spouses) were informed about the study’s purpose and significance. After obtaining written informed consent, paper questionnaires were distributed. Participants completed the questionnaires independently. For participants unable to complete the questionnaires independently, researchers verbally administered the items using standardized instructions and recorded responses accurately. Upon completion, researchers immediately checked questionnaires for missing items and collected them after any necessary additions were made. Two researchers reviewed, coded, and entered the data into Microsoft Excel software. Data entry accuracy was verified using a dual-entry method.

### 2.7 Statistical analysis

Data analysis was performed using SPSS 25.0 and Amos 29.0 software, with the statistical significance level set at *α* = 0.05. Descriptive statistics are presented as frequencies and percentages for categorical data, and as mean ± standard deviation for continuous data. Group comparisons were conducted using independent samples *t*-tests or one-way analysis of variance (ANOVA) when the assumption of homogeneity of variance was met; Welch’s *t*-test was employed when this assumption was violated. Pearson correlation analysis was used to examine bivariate relationships. Multiple linear regression analysis was conducted to identify influencing factors. Prior to the regression analyses, standard diagnostic procedures confirmed that the assumptions of linearity, normality of residuals, homoscedasticity, and absence of influential outliers were adequately met.

The Actor-Partner Interdependence Model (APIM) was constructed using structural equation modeling (SEM). Model parameters were estimated using the maximum likelihood method. Model fit was assessed using the following indices: chi-square to degrees of freedom ratio (χ^2^/df), comparative fit index (CFI), goodness-of-fit index (GFI), adjusted goodness-of-fit index (AGFI), normed fit index (NFI), Tucker-Lewis index (TLI), incremental fit index (IFI), standardized root mean square residual (SRMR), and root mean square error of approximation (RMSEA). The model was considered to have a good fit if the following criteria were met: *χ*^2^/df < 3, CFI > 0.90, GFI > 0.90, AGFI > 0.90, NFI > 0.90, TLI > 0.90, IFI > 0.90, SRMR < 0.08, and RMSEA < 0.08.

To reduce the risk of overfitting and Type I error, covariate selection for the multivariable regression models was guided by both theoretical considerations and statistical criteria. Theoretically informed covariates—including age, education level, number of comorbidities, and number of stents implanted-were selected based on their established associations with benefit finding in the literature. Additional variables that were significant in univariate analyses were retained only if they were consistent with theoretical expectations and did not result in variance inflation factors exceeding 5.0, a commonly accepted threshold for multicollinearity. This combined approach balances theoretical relevance with model parsimony.

## 3 Results

### 3.1 Demographic characteristics

The study comprised 212 dyads of young and middle-aged patients experiencing their first acute myocardial infarction (AMI) and their spouses. The mean age of the patients was (43.11 ± 9.34) years, and the mean age of their spouses was (43.99 ± 9.00) years.

### 3.2 Scores on study measures

The scores for the key measures are presented as follows. Patients reported mean scores of (64.77 ± 13.05) on the BFS, (71.73 ± 14.88) on the CD-RISC, (7.28 ± 2.03) on the FAI, (27.53 ± 8.85) on the SCSQ, with mean item scores of (1.62 ± 0.44) for the Positive Coping subscale and (1.01 ± 0.59) for the Negative Coping subscale. Similarly, spouses reported mean scores of (65.65 ± 12.87) on the BFS, (72.37 ± 13.89) on the CD-RISC, and (7.26 ± 2.05) on the FAI, (28.32 ± 8.71) on the SCSQ, with mean item scores of (1.65 ± 0.42) for Positive Coping and (1.06 ± 0.62) for Negative Coping. The Negative Coping subscale was not included in the subsequent APIM analyses, as it was not significantly associated with benefit finding (see Section 3.4 for details). Based on the BFS theoretical range (22–110) and its midpoint (66 points, corresponding to the scale midpoint of 3 across 22 items), these scores fall within the moderate-low range (see Table 1, Supplementary Table S1).

**Table 1:** Differences in BFS scores by patient and spouse characteristics (*n* = 212).

Characteristic		Cases/Persons (*n*, %)	Patient BFS score (points) (Mean ± SD)	Test statistic	*p*-value	Spouse BFS score (points) (Mean ± SD)	Test statistic	*p*-value
Patient sex	Male	182 (85.8%)	63.60 ± 12.24	−3.272^b^	0.001	64.23 ± 11.75	−4.088^b^	<0.001
	Female	30 (14.2%)	71.83 ± 15.65			74.23 ± 15.98		
Spouse sex	Male	30 (14.2%)	71.80 ± 15.66	3.256^b^	0.001	74.20 ± 11.75	4.071^b^	<0.001
	Female	182 (85.8%)	63.61 ± 12.24			64.24 ± 11.75		
Residence	Rural	114 (53.8%)	59.13 ± 11.06	−7.651^b^	<0.001	61.56 ± 11.17	5.293^b^	<0.001
	Urban	98 (46.2%)	71.33 ± 12.14			70.40 ± 13.13		
Patient employment status	Employed	152 (71.7%)	66.16 ± 12.85	−3.012^b^	0.003	66.38 ± 13.16	−1.104^b^	0.271
	Not employed	60 (28.3%)	61.23 ± 13.10			63.82 ± 12.08		
Patient education level	Primary or below	64 (30.2%)	58.41 ± 9.99	58.608^a^	<0.001	61.06 ± 11.23	24.668^a^	<0.001
	Junior high	52 (24.5%)	59.56 ± 8.99			61.63 ± 9.27		
	Senior high/Technical secondary	35 (16.5%)	60.03 ± 9.44			61.97 ± 10.80		
	College or above	61 (28.8%)	78.61 ± 10.04			75.98 ± 12.58		
Spouse education level	Primary or below	68 (32.1%)	61.15 ± 11.75	18.679^a^	<0.001	59.57 ± 9.99	45.355^a^	<0.001
	Junior high	43 (20.3%)	61.28 ± 10.19			59.02 ± 9.09		
	Senior high/Technical secondary	42 (19.8%)	60.64 ± 9.92			64.55 ± 10.82		
	College or above	59 (27.8%)	74.42 ± 13.05			78.25 ± 10.32		
Medical payment	UEBMI	93 (43.9%)	68.16 ± 12.89	−3.429^b^	0.001	68.72 ± 13.64	−3.138^b^	0.002
	URRBMI	119 (56.1%)	62.12 ± 12.61			63.24 ± 11.74		
Monthly household income (CNY)	≤5,000	12 (5.7%)	62.42 ± 20.12	15.672^a^	<0.001	71.33 ± 21.15	14.231^a^	<0.001
	5,000 ~ 9,999	144 (67.9%)	61.98 ± 10.35			62.55 ± 10.27		
	≥10,000	56 (26.4%)	72.45 ± 14.30			72.38 ± 13.55		
Number of children	0	21 (9.9%)	77.67 ± 13.81	20.451^a^	<0.001	78.38 ± 13.90	17.236^a^	<0.001
	1	94 (44.3%)	67.87 ± 12.85			67.69 ± 12.11		
	≥2	97 (45.8%)	58.98 ± 9.73			60.71 ± 10.38		
Patient comorbidities (n)	0	71 (33.5%)	69.45 ± 16.36	7.400^a^	0.001	69.01 ± 16.00	3.767^a^	0.025
	1	130 (61.3%)	62.56 ± 10.38			64.01 ± 10.68		
	2 ~ 3	11 (5.2%)	60.64 ± 9.70			63.27 ± 10.57		
Spouse comorbidities (n)	0	71 (33.5%)	67.59 ± 15.74	1.296^a^	0.276	69.59 ± 16.25	5.285^a^	0.006
	1	127 (59.9%)	63.71 ± 11.06			63.79 ± 10.50		
	2 ~ 3	14 (6.6%)	60.07 ± 12.92			62.50 ± 12.87		
Stents implanted (n)	1	170 (80.2%)	66.38 ± 12.55	3.715^b^	<0.001	66.34 ± 12.61	1.587^b^	0.114
	≥2	42 (19.8%)	58.26 ± 13.19			62.83 ± 13.67		

BFS, Benefit Finding Scale; UEBMI, Urban Employee Basic Medical Insurance; URRBMI, Urban and Rural Resident Basic Medical Insurance.

a Analysis of Variance (ANOVA) was used.

b Independent samples *t*-test was used.

“Not employed” includes retired and unemployed participants (merged due to small sample sizes: retired patients n = 4, retired spouses *n* = 8).

Monthly Household Income (CNY):≥10,000″ includes the original 10,000–19,999 and ≥20,000 categories (merged due to small sample size: ≥20,000 *n* = 7).

Number of Children: ≥2″ includes the original 2–3 and >3 categories (merged due to small sample size: >3 *n* = 8).

Number of comorbidities and hospitalizations per year were treated as continuous variables in the regression models.

Additional descriptive comparisons for spouse employment status, patient hospitalizations per year, and infarct location are presented in Supplementary Table S1.

### 3.3 Differences in BFS scores by patient and spouse characteristics

### 3.4 Correlations among study variables

Correlations among all study variables are presented in (Table 2). Given the high correlation between patient BFS and CD-RISC (*r* = 0.900) and between patient BFS and SCSQ (*r* = 0.733), multicollinearity diagnostics were performed. Variance Inflation Factor (VIF) values for all predictors in the final APIM were below 5.0 (range: 1.23–4.56), and tolerance values exceeded 0.20, indicating that multicollinearity did not unduly influence the model estimates. Nevertheless, the very high correlations between BFS and CD-RISC (*r* = 0.900 for patients, *r* = 0.866 for spouses) raise concerns about potential construct overlap and common method variance, as all data were self-reported. These findings should therefore be interpreted with caution.

**Table 2:** Correlations among study variables for patients and spouses (*r*-values; *n* = 212).

Variables		Patient				Spouse			
		BFS	CD-RISC	SCSQ	FAI	BFS	CD-RISC	SCSQ	FAI
Patient	BFS	1							
	CD-RISC	0.900^**^	1						
	SCSQ	0.733^**^	0.782^**^	1					
	FAI	0.659^**^	0.666^**^	0.520^**^	1				
Spouse	BFS	0.709^**^	0.649^**^	0.579^**^	0.498^**^	1			
	CD-RISC	0.669^**^	0.702^**^	0.629^**^	0.522^**^	0.866^**^	1		
	SCSQ	0.591^**^	0.634^**^	0.726^**^	0.425^**^	0.693^**^	0.772^**^	1	
	FAI	0.415^**^	0.402^**^	0.339^**^	0.594^**^	0.625^**^	0.623^**^	0.471^**^	1

** indicates statistical significance at *p* < 0.01.

Negative coping was not significantly correlated with BFS for patients (*r* = −0.112, *p* = 0.105) or spouses (*r* = −0.098, *p* = 0.156), nor was it significantly associated with the other key study variables. Given its lack of association with benefit finding and to maintain model parsimony, negative coping was not included in the subsequent APIM. The present analyses therefore focused on positive coping as the theoretically relevant adaptive coping dimension.

### 3.5 Multivariate analysis of factors associated with benefit finding

Benefit finding scores for both young and middle-aged first-episode AMI patients and their spouses served as the dependent variables. Prior to the regression analyses, diagnostic tests confirmed that the assumptions of linearity, normality of residuals, homoscedasticity, and absence of influential outliers were adequately met (see Section 2.7 for details). Separate multiple linear regression models were constructed for patients and spouses. Covariates were selected based on a combination of theoretical relevance and univariate significance, as described in Section 2.7 (see Tables 3, 4 for detailed variable coding).

**Table 3:** Factors associated with patient benefit finding (*n* = 212).

Characteristic	*β* (unstandardized coefficient)	SE (standard error)	β* (standardized coefficient)	*t* value	*p*-value
Intercept	16.419	5.358	—	3.064	0.003
Patient education level (college or above)	5.025	1.896	0.175	2.650	0.009
Patient comorbidities (n)	−3.368	0.863	−0.126	−3.905	<0.001
Patient CD-RISC Score	0.491	0.060	0.559	8.232	<0.001
Patient positive coping strategies	0.359	0.148	0.147	2.417	0.017
Stents Implanted (n)	−2.640	1.010	−0.120	−2.610	0.010
Spousal CD-RISC Score	2.800	1.120	0.150	2.500	0.013

*R* = 0.929, *R*^2^ = 0.864, Adjusted *R*^2^ = 0.841, *F* = 38.260, *p* < 0.001. Variable coding: Gender: Male = 1, Female = 2; Residence: Rural = 1, Urban = 2; Education: Primary or below = 1, Junior high = 2, Senior high/Technical secondary = 3, College or above = 4; Employment: Employed = 1, Not employed = 0 (original Retired and Unemployed categories merged due to small sample sizes); Medical insurance: URRBMI = 1, UEBMI = 2; Monthly household income (CNY): ≤5,000 = 1, 5,000–9,999 = 2, ≥10,000 = 3 (original 10,000–19,999 and ≥20,000 categories merged due to small sample sizes); Number of children: 0 = 0, 1 = 1, ≥2 = 2 (original 2–3 and >3 categories merged due to small sample sizes); Number of comorbidities and hospitalizations per year were treated as continuous variables in regression models; Stents implanted: 1 = 1, ≥2 = 2.

**Table 4:** Factors associated with spouse benefit finding (*n* = 212).

Characteristic	*β* (unstandardized coefficient)	SE (standard error)	β* (standardized coefficient)	*t* value	*p*-value
Intercept	15.668	7.133	—	2.197	0.029
Patient comorbidities (n)	−2.382	1.006	−0.088	2.368	0.019
Spousal education level (college or above)	3.462	1.711	0.121	2.023	0.045
Patient age	0.199	0.086	0.144	2.299	0.023
Spousal age	−0.239	0.086	−0.167	−2.768	0.006
Spousal CD-RISC score	0.475	0.068	0.513	7.013	<0.001
Spousal FAI score	0.720	0.307	0.114	2.342	0.020

*R* = 0.914, *R*^2^ = 0.836, Adjusted *R*^2^ = 0.808, *F* = 29.584, *p* < 0.001. Variable coding same as Table 3.

### 3.6 Path analysis of the actor-partner interdependence model

An Actor-Partner Interdependence Model (APIM) was constructed using the CD-RISC, FAI, and SCSQ scores of both young and middle-aged first-episode AMI patients and their spouses as predictor variables, with their respective BFS scores as outcome variables.

The initial fully saturated model included all theoretically plausible actor and partner paths from resilience, positive coping, and family function to both own and partner benefit finding. This model demonstrated poor fit: *χ*^2^ = 148.52, df = 24, *χ*^2^/df = 6.188, CFI = 0.872, GFI = 0.901, AGFI = 0.812, NFI = 0.843, TLI = 0.821, IFI = 0.875, SRMR = 0.112, RMSEA = 0.157. Modification indices indicated that fit would be substantially improved by removing non-significant paths and allowing the residuals of patient and spouse BFS to correlate.

The removed paths included: patient positive coping → spouse BFS, spouse positive coping → patient BFS, patient FAI → spouse BFS, spouse FAI → patient BFS, patient FAI → patient BFS, patient positive coping → spouse FAI, and patient CD-RISC → spouse FAI (all *p* > 0.05). These paths lacked strong theoretical justification in the AMI dyadic literature. This model refinement should therefore be considered exploratory; the resulting parsimonious model requires cross-validation in future independent samples. After these modifications, the final model showed excellent fit: *χ*^2^ = 23.75, df = 16, *χ*^2^/df = 1.484, CFI = 0.997, GFI = 0.984, AGFI = 0.945, NFI = 0.942, TLI = 0.993, IFI = 0.992, SRMR = 0.032, RMSEA = 0.048 (90% CI: 0.000–0.086).

**Figure 1:**
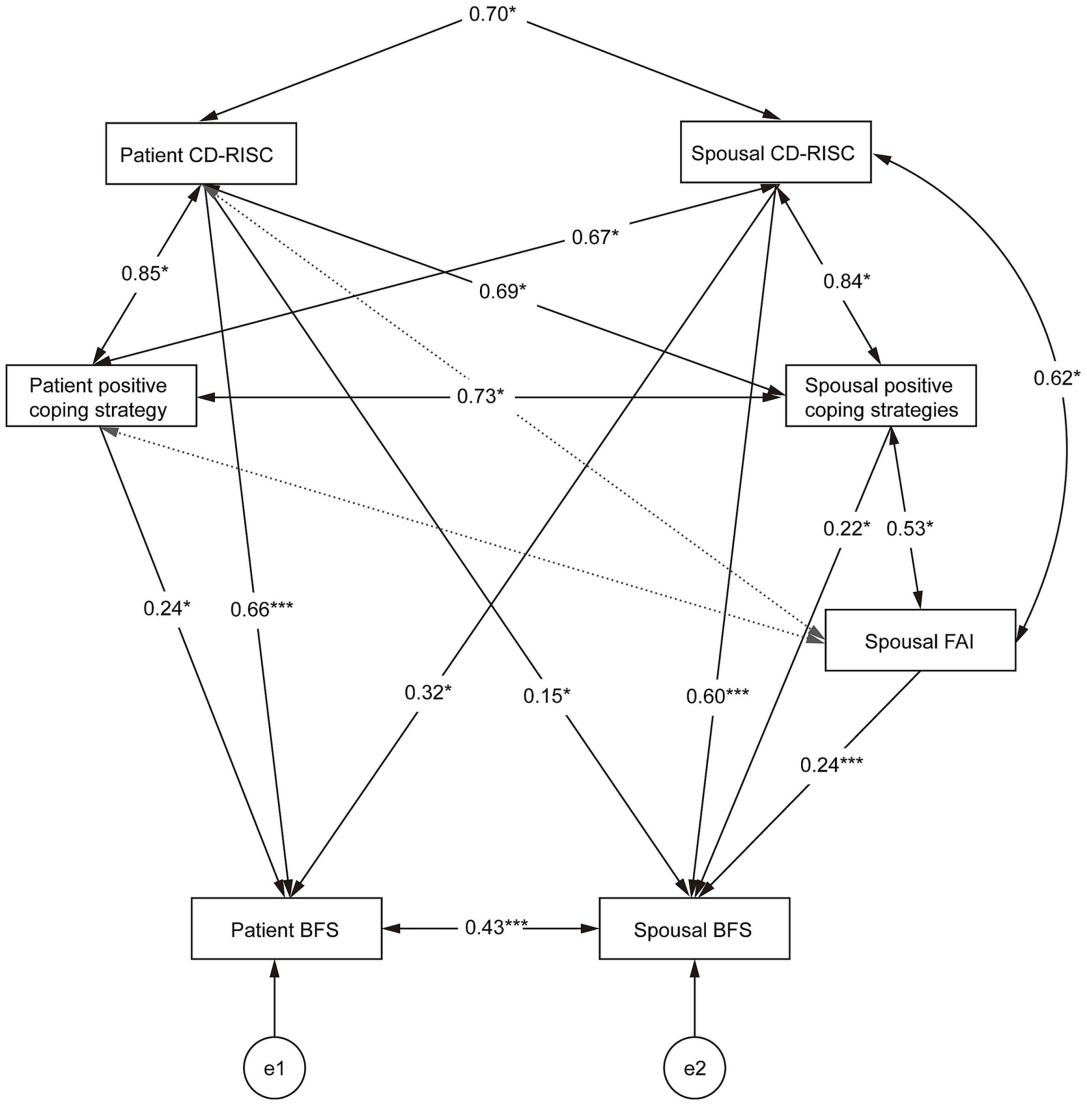
Final actor-partner interdependence model with standardized path coefficients for resilience, positive coping, family function, and benefit finding in patients and spouses. Solid lines represent significant paths, and dotted lines represent non-significant paths. *p < 0.05, **p < 0.01, ***p < 0.001.

All path coefficients reported are standardized estimates (*β*); 95% confidence intervals were derived from maximum likelihood standard errors. Bivariate correlations and covariances among predictor variables—as well as the two non-significant cross-predictor paths involving spouse FAI—are reported in Supplementary Table S2 and are not interpreted as actor or partner effects. The final model with standardized path coefficients is presented in Figure 1, and significant paths with full statistics are reported in Table 5.

**Table 5:** Standardized path coefficients for the final APIM.

Path	Β (Standardized)	SE	95%CI	*p*-value
Actor effects on benefit finding				
Patient CD-RISC score → patient BFS score	0.66	0.049	[0.56, 0.76]	<0.001
Patient positive coping strategies score → patient BFS score	0.24	0.126	[0.02, 0.46]	0.030
Spousal CD-RISC score → spousal BFS score	0.60	0.053	[0.48, 0.72]	<0.001
Spousal positive coping strategies score → spousal BFS score	0.22	0.148	[0.01, 0.43]	0.042
Spousal FAI score → spousal BFS score	0.24	0.240	[0.10, 0.38]	0.001
Partner effects on benefit finding				
Patient CD-RISC score → spousal BFS score	0.15	0.040	[0.01, 0.29]	0.046
Spousal CD-RISC score → patient BFS score	0.32	0.041	[0.05, 0.59]	0.022
Residual covariance				
Patient BFS Score ↔ Spousal BFS Score	0.43	0.089	[0.25, 0.61]	<0.001
Non-significant paths (removed from final model)				
Patient positive coping strategies score → spouse BFS score	—	—	—	0.242
Spouse positive coping strategies score → patient BFS Score	—	—	—	0.561
Patient FAI score → spousal BFS score	—	—	—	0.784
Spousal FAI score → patient BFS score	—	—	—	0.225
Patient FAI score → patient BFS score	—	—	—	0.659

Benefit finding *R*^2^: 0.816 (Patients); 0.768 (Spouses). All coefficients are standardized. 95% CIs are based on standard errors from maximum likelihood estimation. Bivariate correlations among predictor variables are reported in Supplementary Table S2. For paths removed from the final model, standardized estimates were not retained; *p*-values are reported for transparency.

## 4 Discussion

### 4.1 Moderate-low levels of benefit finding

The results indicate that both patients and spouses reported benefit finding scores in the moderate-low range, suggesting significant room for improvement. These findings align with studies involving stroke patients (Yin et al., 2025) and spouses of type 2 diabetes patients (Wu et al., 2025). AMI, as a sudden and highly life-threatening event, imposes significant psychological distress on both patients and spouses, potentially hindering their ability to perceive positive meaning from the illness experience (Zhou et al., 2020). Furthermore, as young and middle-aged individuals often serve as primary financial providers for their families, the career disruption, economic burden, and long-term treatment pressures associated with the illness may further suppress the development of benefit finding (Zhang Q. et al., 2024). Healthcare professionals should therefore prioritize early attention to the psychological adaptation of both patients and their spouses, guiding them to reassess life priorities and the value of family relationships.

### 4.2 Actor effects: individual psychological resources as foundations of benefit finding

A central finding of this study, derived from the APIM analysis, is the robust actor effects observed for both patients and spouses. Specifically, each individual’s own psychological resilience and positive coping style significantly predicted their own benefit finding. However, given the high bivariate correlations between resilience and benefit finding, the strength of these associations may be inflated by construct overlap; thus, these results should be interpreted as preliminary. These results align with the broader benefit finding literature and provide novel evidence within the AMI dyadic context (Chen et al., 2022).

The strong actor effect of resilience suggests that the capacity to adaptively perceive and respond to illness-related challenges serves as a foundational psychological resource for deriving positive meaning. Patients with higher resilience are better equipped to confront the abrupt life changes imposed by AMI and to reconstruct a coherent, growth-oriented illness narrative. Similarly, resilient spouses can draw upon internal resources to navigate the complexities of caregiving while maintaining psychological equilibrium, which in turn enables them to recognize caregiving-related growth. The significant actor effect of positive coping further reinforces this interpretation: individuals who adopt active, problem-focused coping strategies are more likely to reframe the illness experience in ways that facilitate benefit finding (Wang et al., 2020).

Notably, for spouses, family function also exhibited a significant actor effect on their own benefit finding, consistent with research involving other caregiving populations (Liu et al., 2019). This suggests that spouses’ subjective satisfaction with family cohesion, emotional exchange, and mutual support constitutes an important resource that can be mobilized to foster benefit finding. When spouses perceive strong family function, they can actively communicate with family members, express difficulties, and engage in mutual support, which maximizes family resources and alleviates caregiving burden (Sidek et al., 2022). Healthcare professionals should therefore consider comprehensive interventions that simultaneously strengthen multiple levels of individual resources—resilience, adaptive coping, and perceived family functioning—delivered not only to patients but also to their spousal.

### 4.3 Partner effects and dyadic patterns: the interdependence of resilience within the couple

A key contribution of this study is the demonstration of significant partner effects of psychological resilience on benefit finding within the APIM framework. Patients’ resilience positively predicted spouses’ benefit finding, and spouses’ resilience positively predicted patients’ benefit finding. These cross-partner effects, directly demonstrated by our data, provide evidence for the interdependent nature of psychological adaptation following AMI and extend prior APIM-based research in other health contexts (Zhang Z. Z. et al., 2024) to the AMI population.

The dyadic patterns testing further illuminates the nature of this interdependence. The mixed pattern observed for patients—where the partner effect of spousal resilience on patient benefit finding is approximately half the magnitude of the patient’s own actor effect—indicates that patients’ benefit finding is shaped by both their own resilience and their spouse’s resilience. A cautious interpretation of this finding is that patients may be particularly receptive to spousal influence during the acute recovery phase. It is plausible that a spouse’s calm and competent navigation of medical crises could help reduce the patient’s illness-related uncertainty, creating a supportive psychological environment that is conducive to benefit finding. However, this proposed “resilience buffer” mechanism is speculative and should be viewed as a hypothesis requiring empirical validation in longitudinal studies. Conversely, the predominantly actor-driven pattern for spouses indicates that while spouses do benefit from their partner’s resilient behavior, their own internal coping resources remain the primary driver of their benefit finding. This observed asymmetry is consistent with the role dynamics inherent in caregiving relationships, although the causal pathways underlying this pattern cannot be determined from cross-sectional data alone.

These findings carry implications for clinical practice while also highlighting directions for future research. The data directly show that patients’ and spouses’ resilience are mutually associated with each other’s benefit finding, which suggests that interventions targeting only one member of the dyad may be insufficient. Dyadic interventions—such as collaborative resilience training, joint reflective exercises on shared illness experiences, and intimacy enhancement programs (Liu et al., 2023)—represent promising strategies that warrant investigation. However, whether improvements in one partner’s resilience causally lead to enhanced benefit finding in the other remains an open question that should be tested in future intervention studies with longitudinal or experimental designs.

### 4.4 Supplementary factors shaping benefit finding through the dyadic lens

Beyond the core APIM findings, certain sociodemographic and clinical factors identified warrant interpretation through the dyadic lens, as they may contextualize the actor and partner pathways described above.

For patients, higher educational attainment, fewer comorbidities, and fewer implanted stents were associated with greater benefit finding. Higher education facilitates access to disease-related information and cognitive reframing, enabling more adaptive transformation of negative experiences. Multiple chronic conditions impose a cumulative disease management burden that may deplete the psychological resources necessary for benefit finding, while requiring two or more stents can trigger perceptions of “premature aging” among young and middle-aged patients (Yin et al., 2025). From a dyadic perspective, when the patient’s clinical situation is more complex, the spouse’s caregiving burden intensifies, potentially constraining their availability to serve as an effective resilience model for the patient. Healthcare professionals should therefore prioritize patients with lower education levels through simplified health education tools, while strengthening case management and integrated chronic disease care resources for those with multimorbidity (Li et al., 2023).

For spouses, younger age, higher education, older patient age, and fewer patient comorbidities predicted higher benefit finding. Higher-educated spouses may better comprehend disease management complexities and utilize diverse information channels, reducing caregiving uncertainty. As spouses age, their own risk for chronic conditions increases, and combined with caregiving demands, this heightens their vulnerability to exhaustion, making it difficult to focus on positive experiences. Older patients often have adult children involved in care, allowing the spouse to transition from sole caregiver to care coordinator, thereby preserving psychological space to recognize positive aspects. Conversely, multimorbidity in the patient intensifies the spouse’s caregiving burden and may precipitate anticipatory grief (Wen et al., 2022). These findings reinforce that dyadic interventions must be individually tailored, taking into account each member’s personal characteristics and their partner’s clinical profile.

### 4.5 Limitations

Several limitations should be acknowledged. First, the high correlations between benefit finding and resilience (*r* = 0.900 for patients, *r* = 0.866 for spouses) suggest potential construct overlap and common method variance, as all measures were self-reported. Although multicollinearity diagnostics were within acceptable limits, future research should employ multi-method assessments to reduce common method bias. Second, the single-center, cross-sectional design precludes causal inferences. The proposed mechanisms—such as the resilience buffer hypothesis and cascading dyadic effects—should be considered preliminary and require validation through longitudinal or intervention studies. Additionally, the APIM modifications were exploratory rather than strictly theory-driven, and the resulting model requires cross-validation in independent samples. The 95% confidence intervals for path coefficients were derived from standard errors of maximum likelihood estimation rather than bootstrapping; future studies should consider bootstrap methods for more robust inference. Third, the exclusive reliance on quantitative methods limits a comprehensive understanding of the meaning-making processes underlying benefit finding; future research should integrate qualitative approaches. Finally, although covariate selection was theoretically informed, it was also partially data-driven, which may increase the risk of overfitting. Future studies with larger samples should employ fully theory-driven model specification or regularization techniques.

## 5 Conclusion

This study demonstrates that young and middle-aged patients with first-episode AMI and their spouses experience benefit finding at a moderate-to-low level. Within the APIM framework, psychological resilience and positive coping exhibited robust actor effects on own benefit finding for both dyad members, while family function was a significant actor effect specifically for spouses. Crucially, the resilience of each dyad member exerted a positive partner effect on the other’s benefit finding, with patients showing a mixed dyadic pattern and spouses a primarily actor-driven pattern. These findings suggest that healthcare professionals should adopt a dyadic perspective, implementing interventions that simultaneously enhance resilience and positive coping capacity in both patients and spouses to facilitate higher levels of benefit finding in the aftermath of AMI.

## Data Availability

The original contributions presented in the study are included in the article/Supplementary material, further inquiries can be directed to the corresponding author.

## References

[ref1] Antoni M. H. Lehman J. M. Kilbourn K. M. Boyers A. E. Culver J. L. Alferi S. M. . (2001). Cognitive-behavioral stress management intervention decreases the prevalence of depression and enhances benefit finding among women under treatment for early-stage breast cancer. Health Psychol. 20, 20–32. doi: 10.1037//0278-6133.20.1.20 11199062

[ref2] Brand C. Barry L. Gallagher S. (2016). Social support mediates the association between benefit finding and quality of life in caregivers. J. Health Psychol. 21, 1126–1136. doi: 10.1177/1359105314547244, 25205775

[ref3] Cai Q. Z. Qu Y. S. Zhang J. (2020). Analysis of the relationship between family function and depressive mood in primary caregivers of patients with coronary heart disease. J. Clin. Nurs. 19, 10–13. doi: 10.3969/j.issn.1671-8933.2020.01.003

[ref4] Chen C. Chen Y. Y. Liu X. Y. Gu W. N. Fan G. H. Zhu P. T. (2022). Construction of a structural equation model of influencing factors of benefit finding in patients with lung cancer. Mil. Nurs. 39, 39–43. doi: 10.3969/j.issn.1008-9993.2022.06.010

[ref5] Connor K. M. Davidson J. R. (2003). Development of a new resilience scale: the Connor-Davidson resilience scale (CD-RISC). Depress. Anxiety 18, 76–82. doi: 10.1002/da.10113, 12964174

[ref6] Ibanez B. James S. Agewall S. Antunes M. J. Bucciarelli-Ducci C. Bueno H. . (2018). 2017 ESC guidelines for the management of acute myocardial infarction in patients presenting with ST-segment elevation: the task force for the management of acute myocardial infarction in patients presenting with ST-segment elevation of the European Society of Cardiology (ESC). Eur. Heart J. 39, 119–177. doi: 10.1093/eurheartj/ehx393, 28886621

[ref7] Jin X. M. Tang L. P. Cao Y. Li T. (2021). Current status and influencing factors of benefit finding in patients undergoing urinary diversion and abdominal wall ostomy for bladder cancer. Chin. Nurs. Res. 35, 2425–2430. doi: 10.12102/j.issn.1009-6493.2021.13.032

[ref8] Kenny D. A. West T. V. Malloy T. E. Albright L. (2006). Componential analysis of interpersonal perception data. Personal. Soc. Psychol. Rev. 10, 282–294. doi: 10.1207/s15327957pspr1004_1, 17201589

[ref9] Lee Y. Lin P. Y. Chien C. Y. Fang F. M. Wang L. J. (2018). A comparison of psychological well-being and quality of life between spouse and non-spouse caregivers in patients with head and neck cancer: a 6-month follow-up study. Neuropsychiatr. Dis. Treat. 14, 1697–1704. doi: 10.2147/ndt.S162116, 29988736 PMC6029606

[ref10] Li L. P. Rao D. F. Chen X. X. Qi X. Y. Chen X. X. Wang X. Q. . (2023). The impact of hospital-family integrated continuation nursing based on information technology on patients unhealthy mood, family function and sexual function after cervical cancer surgery. Medicine (Baltimore) 102:e33504. doi: 10.1097/md.0000000000033504, 37083787 PMC10118314

[ref11] Liu Z. Z. Liu M. J. Zhang L. F. (2014). A concept analysis of benefit finding in breast cancer patients. Chin. J. Pract. Nurs. 30, 17–20. doi: 10.3760/cma.j.issn.1672-7088.2014.13.005

[ref12] Liu Y. N. Xie H. N. Zhang J. M. (2019). Correlation between family care and quality of life in elderly patients with hypertension. Int. J. Nurs. 38, 892–895. doi: 10.3760/cma.j.issn.1673-4351.2019.07.009

[ref13] Liu Z. W. Zhang Z. X. Mei Y. X. Chen S. Y. Lin B. L. (2023). Research progress on couple-based illness communication interventions in cancer patients. Chin. J. Nurs. 58, 2288–2293. doi: 10.3761/j.issn.0254-1769.2023.18.017

[ref14] McHorney C. A. Mansukhani S. G. Anatchkova M. Taylor N. Wirtz H. S. Abbasi S. . (2021). The impact of heart failure on patients and caregivers: a qualitative study. PLoS One 16:e0248240. doi: 10.1371/journal.pone.0248240, 33705486 PMC7951849

[ref15] Rao S. V. O'Donoghue M. L. Ruel M. Rab T. Tamis-Holland J. E. Alexander J. H. . (2025). 2025 ACC/AHA/ACEP/NAEMSP/SCAI guideline for the management of patients with acute coronary syndromes. J. Am. Coll. Cardiol. 85, 2135–2237. doi: 10.1016/j.jacc.2024.11.009, 40013746

[ref16] Sidek N. N. Kamalakannan S. Tengku Ismail T. A. Musa K. I. Ibrahim K. A. Abdul Aziz Z. . (2022). Experiences and needs of the caregivers of stroke survivors in Malaysia-a phenomenological exploration. Front. Neurol. 13:996620. doi: 10.3389/fneur.2022.996620, 36212650 PMC9539245

[ref17] Smilkstein G. Ashworth C. Montano D. (1982). Validity and reliability of the family APGAR as a test of family function. J. Fam. Pract. 15, 303–311. 7097168

[ref18] Sun C. Y. Lin Z. Zhou M. J. Gu Z. J. Wang M. Gu J. Y. . (2022). Current status and influencing factors of benefit finding in patients with inflammatory bowel disease. J. Nurs. 29, 1–6. doi: 10.16460/j.issn1008-9969.2022.06.001

[ref19] Tang Q. Bai X. L. Lou T. (2023). A qualitative study on the psychosocial adaptation experience of young and middle-aged patients with acute myocardial infarction at different stages. J. Nurs. Train. 38, 1915–1919. doi: 10.16821/j.cnki.hsjx.2023.20.019

[ref20] Uchmanowicz I. Faulkner K. M. Vellone E. Siennicka A. Szczepanowski R. Olchowska-Kotala A. (2022). Heart failure care: testing dyadic dynamics using the actor-partner interdependence model (APIM)-a scoping review. Int. J. Environ. Res. Public Health 19:1919. doi: 10.3390/ijerph19041919, 35206131 PMC8871794

[ref21] Wang Z. Ma L. Liu M. Fan J. Hu S. (2023). Summary of the 2022 report on cardiovascular health and diseases in China. Chin. Med. J. 136, 2899–2908. doi: 10.1097/cm9.0000000000002927, 38018129 PMC10752444

[ref22] Wang W. N. Zhang Z. X. Mei Y. X. Zhang L. Y. Guo E. F. (2020). Development of stress and coping theory and its application in intervention research on caregivers of chronic diseases. Mod. Prev. Med. 47, 75–78. doi: 10.20043/j.cnki.mpm.2020.01.020

[ref23] Wei R. Q. Liao D. Deng T. Liu H. Chen Y. (2019). Study on the current status and influencing factors of caregiver burden among primary caregivers of brain tumor patients. J. Nurs. Adm. 19, 710–714. doi: 10.3969/j.issn.1671-315x.2019.10.006

[ref24] Wen X. M. Cai P. Chu J. H. Su H. Tong T. S. Gu D. F. (2022). Application of Satir model intervention in family caregivers of patients with esophageal cancer. Chin. Nurs. Res. 36, 1671–1675. doi: 10.12102/j.issn.1009-6493.2022.09.032

[ref25] Wu N. Meng L. X. Ji H. Y. Li S. Q. Feng C. Q. (2025). Current status and influencing factors of benefit finding among caregivers of patients with type 2 diabetes mellitus. Nurs. Pract. Res 22, 100–107. doi: 10.3969/j.issn.1672-9676.2025.01.015

[ref26] Wu L. Tan Y. Liu Y. (2017). Factor structure and psychometric evaluation of the Connor-Davidson resilience scale in a new employee population of China. BMC Psychiatry 17:49. doi: 10.1186/s12888-017-1219-0, 28152997 PMC5290619

[ref27] Xie Y. N. (1998). A preliminary study on the reliability and validity of the simplified coping style scale. Chin. J. Clin. Psych. 02, 53–54. doi: 10.16128/j.cnki.1005-3611.1998.02.018

[ref28] Yin M. J. Chen L. F. Yang X. J. Hu X. L. (2025). Current status and influencing factors of benefit finding in young and middle-aged stroke patients. J. Nurs. Rehabil. 24, 1–12. doi: 10.3969/j.issn.1671-9875.2025.02.001

[ref29] Zhang Z. Z. Li X. Wang Z. Yang Y. T. Zhu D. G. Jiang X. G. . (2024). Influencing factors and path analysis of benefit finding in cervical cancer patients and their spouses. Chin. J. Nurs. 59, 2214–2221. doi: 10.3761/j.issn.0254-1769.2024.18.005

[ref30] Zhang Q. Ning L. Yang X. Yu M. Zheng B. Wang Y. . (2024). Return to work experience of young and middle-aged patients with acute myocardial infarction: a longitudinal qualitative study. J. Cardiovasc. Nurs. 39, 465–476. doi: 10.1097/jcn.0000000000001019, 37639559

[ref31] Zhou H. J. Liang Z. J. Zhong M. R. Ma P. (2020). Current status and influencing factors of social function in young and middle-aged patients after percutaneous coronary intervention for coronary heart disease. Chin. J. Mod. Nurs. 26, 1025–1031. doi: 10.3760/cma.j.cn115682-20191107-04059

