# Supplementary material for: Influencing factors and pathways of benefit finding in young and middle-aged patients with first-episode acute myocardial infarction and their spouses: a path analysis
Source: Front Psychol. 2026 Jul 1;17:1852033. doi: 10.3389/fpsyg.2026.1852033 (PMC13368501; doi:10.3389/fpsyg.2026.1852033)
Supplement: Supplementary file 1 [file Table_1.DOCX]

**Table S1 Supplementary Descriptive Comparisons for Variables Not Included in the Main Table (n = 212)**

| *Characteristic* | | *Cases/*  *Persons (n, %)* | *Patient BFS Score (points)*  *(Mean±SD)* | *Test Statistic* | *P-value* | *Spouse BFS Score (points)*  *(Mean±SD)* | *Test Statistic* | *P-value* |
| --- | --- | --- | --- | --- | --- | --- | --- | --- |
| Spouse Employment Status | Employed | 175（82.5%） | 64.27±12.73 | -0.988 *^b^* | 0.324 | 64.95±11.95 | -1.521*^b^* | 0.130 |
|  | Not employed | 37（17.5%） | 67.14±14.61 |  |  | 69.03±17.05 |  |  |
| Patient Hospitalizations/year (n) | 0 | 71（33.5%） | 65.51±16.03 | 3.043*^a^* | 0.050 | 66.14±14.55 | 0.885*^a^* | 0.414 |
|  | 1 | 128（60.4%） | 64.92±11.44 |  |  | 65.84±12.19 |  |  |
|  | 2~4 | 13（6.1%） | 59.23±8.66 |  |  | 61.08±8.94 |  |  |
| Infarct Location | Left Coronary Artery (LCA) | 105（49.5%） | 64.05±13.50 | -0.796*^b^* | 0.427 | 65.52±13.36 | -0.137*^b^* | 0.891 |
|  | Right Coronary Artery (RCA) | 107（50.5%） | 65.48±12.63 |  |  | 65.77±12.44 |  |  |

**Notes:** 1.a: Analysis of Variance (ANOVA) was used. b: Independent samples t-test was used.

2. "Not employed" includes retired and unemployed participants (merged due to small sample sizes: retired patients n = 4, retired spouses n = 8).
