# Supplementary material for: Influencing factors and pathways of benefit finding in young and middle-aged patients with first-episode acute myocardial infarction and their spouses: a path analysis
Source: Front Psychol. 2026 Jul 1;17:1852033. doi: 10.3389/fpsyg.2026.1852033 (PMC13368501; doi:10.3389/fpsyg.2026.1852033)
Supplement: Supplementary file 2 [file Table_2.DOCX]

**Table S2 Correlations and Covariances Among Predictor Variables**

| *Association* | *Β*  *（Standardized）* | *SE* | *95%CI* | *P-value* |
| --- | --- | --- | --- | --- |
| Patient CD-RISC Score ↔ Spousal CD-RISC Score | 0.70 | 0.311 | [0.09, 1.31] | 0.024 |
| Patient CD-RISC Score ↔ Patient positive coping strategies Score | 0.85 | 0.335 | [0.19, 1.51] | 0.011 |
| Spousal CD-RISC Score ↔ Spousal positive coping strategies Score | 0.84 | 0.352 | [0.15, 1.53] | 0.017 |
| Patient CD-RISC Score ↔ Spousal positive coping strategies Score | 0.69 | 0.301 | [0.10, 1.28] | 0.022 |
| Patient positive coping strategies Score ↔ Spousal CD-RISC Score | 0.67 | 0.332 | [0.02, 1.32] | 0.044 |
| Patient positive coping strategies Score ↔ Spousal positive coping strategies Score | 0.73 | 0.326 | [0.09, 1.37] | 0.025 |
| Spousal CD-RISC Score ↔ Spousal FAI Score | 0.62 | 0.297 | [0.04, 1.20] | 0.037 |
| Spousal positive coping strategies Score ↔ Spousal FAI Score | 0.53 | 0.260 | [0.02, 1.04] | 0.041 |
| Patient positive coping strategies Score ↔ Spousal FAI Score | 0.40 | 0.072 | [0.26, 0.54] | 0.092 |
| Patient CD-RISC Score ↔ Spousal FAI Score | 0.40 | 0.217 | [0.03, 0.83] | 0.065 |

**Note: These associations represent correlations or covariances among predictor variables and are not directional actor or partner effects on benefit finding. All coefficients are standardized.**
